# Effects of Elastic-Band Resistance and Plyometric Training on Serum MMP-2, MMP-9, TIMPs and Coenzyme Q10 in Collegiate Tennis Players: An Exploratory, Non-Randomised Three-Arm Controlled Study

**DOI:** 10.3390/metabo16100801

**Published:** 2026-10-21

**Authors:** Emine Büyükkaya, Salih Öner, Yavuz Yasul, Hamit Hakan Alp, Vedat Çınar, Beyza Ertan, Mustafa Karadağ

**Affiliations:** 1 Department of Physical Education and Sports, Faculty of Sport Sciences, Van Yuzuncu Yil University, 65090 Van, Türkiye; buyukkayaemine00@gmail.com; 2 Bafra Vocational School, Ondokuz Mayıs University, 55400 Samsun, Türkiye; yavuz.yasul@omu.edu.tr; 3 Department of Biochemistry, Faculty of Medicine, Van Yuzuncu Yil University, 65090 Van, Türkiye; hhakan.alp@yyu.edu.tr; 4 Department of Physical Education and Sports, Faculty of Sport Sciences, Fırat University, 23119 Elazığ, Türkiye; vcinar@firat.edu.tr (V.Ç.); mkaradag@firat.edu.tr (M.K.); 5 Department of Coaching Education, Faculty of Sport Sciences, Van Yuzuncu Yil University, 65090 Van, Türkiye; beyzaoge@yyu.edu.tr

**Keywords:** tennis, elastic resistance training, plyometric exercise, extracellular matrix, tissue inhibitor of metalloproteinases, ubiquinone, antioxidants

## Abstract

Background/Objectives: Elastic-band resistance and plyometric training, which differ in eccentric and impact loading, are commonly added to tennis conditioning, but their effects on circulating markers related to extracellular-matrix turnover and antioxidant status are unknown. Methods: In this exploratory, matched, non-randomised controlled study, 24 collegiate tennis players (11 women, 13 men) were allocated to habitual tennis training only (CON) or to 8 weeks of additional elastic-band resistance (RBT) or plyometric training (PLY); 23 (CON 8, RBT 7, PLY 8) were analysed. Fasting serum MMP-2, MMP-9, TIMP-1, TIMP-2 and coenzyme Q10 (CoQ10) were measured by enzyme-linked immunosorbent assay (ELISA) approximately 24 h after the final session and compared by baseline-adjusted analysis of covariance with multiplicity control and sensitivity analyses. Results: Baseline-adjusted CoQ10 was lower in RBT than in CON (Hedges’ g −0.86, 95% CI −1.47 to −0.26) and PLY (g −0.80, 95% CI −1.48 to −0.12); in RBT, CoQ10 fell from 16.6 ± 4.3 to 8.3 ± 1.9 ng/mL. Adjusted TIMP-1 was lower in both training groups than in CON, reflecting an increase in CON rather than a decrease with training. TIMP-2 was lower in RBT than in CON and PLY (g −1.00 and −1.10), but this was not confirmed by rank-based analysis. MMP-9 fell over time in all groups without a group effect, and MMP-2 was unchanged. Conclusions: In this small, non-randomised study, adding elastic-band training to tennis practice was associated with lower serum CoQ10 and, less robustly, TIMP-2; plyometric training was not associated with changes beyond those seen in all groups, although only very large effects were detectable. These hypothesis-generating findings require confirmation in adequately powered randomised trials using chromatographic CoQ10 measurement.

## 1. Introduction

Tennis is an intermittent sport in which repeated accelerations, decelerations, changes in direction and high-velocity strokes are performed for 90 min or longer, so competitive success depends on a combination of speed, agility, muscular strength and power alongside a well-developed aerobic base [1,2]. Strength and power training is therefore a routine component of tennis conditioning: it increases stroke velocity, improves movement efficiency and is widely believed to reduce injury risk [3,4]. Two modalities are particularly popular because they require minimal equipment and can be embedded into on-court sessions. Elastic-band (Thera-Band) resistance training loads muscle through the full range of motion with variable, ascending tension, produces strength gains similar to those of conventional resistance training [5] and is used to develop rotator-cuff, trunk and hip strength [6], whereas plyometric training exploits the stretch–shortening cycle through rapid eccentric-to-concentric transitions in jumps and medicine-ball throws to enhance rate of force development [7,8] and improves serve velocity, sprint, power and agility in tennis players [9]. Both improve neuromuscular performance in racket-sport athletes [10], but they differ in their mechanical stimulus: band exercises impose relatively low, continuous tension with little eccentric overload or impact, whereas plyometric jumps and throws impose high-velocity eccentric loading and ground-reaction impacts that can transiently induce muscle damage and an acute inflammatory response [11]. Whether these different loading profiles are reflected in the connective-tissue and redox systems that contribute to tissue tolerance to training remains largely unexplored.

Skeletal muscle, tendon and the perimysial extracellular matrix (ECM) adapt to mechanical loading through a tightly regulated cycle of degradation and synthesis [12]. The matrix metalloproteinases (MMPs) are zinc-dependent endopeptidases that initiate this cycle; the gelatinases MMP-2 and MMP-9 degrade type IV collagen, denatured fibrillar collagens and basement-membrane components, thereby permitting satellite-cell migration, angiogenesis and the release of matrix-bound growth factors [13,14]. Their activity is restrained by the tissue inhibitors of metalloproteinases (TIMPs); TIMP-1 preferentially inhibits MMP-9, whereas TIMP-2 both inhibits MMP-2 and, paradoxically, is required for its MT1-MMP-mediated activation [15]. The MMP/TIMP ratio has therefore been used as a surrogate of proteolytic balance, and a chronic shift toward proteolysis has been linked to muscle damage, tendinopathy and cardiovascular remodelling [16]. Circulating MMPs and TIMPs, however, derive from many tissues, and immunoassays measure total (pro- and active) protein rather than enzymatic activity, so serum concentrations cannot be equated with local matrix remodelling in muscle or tendon. Acute exercise transiently increases MMP-9 and, to a lesser extent, MMP-2 in skeletal muscle and the circulation [17,18], but the effects of weeks of training are inconsistent: 8 weeks of callisthenic, but not resistance, training augmented the serum MMP-9 response to acute resistance exercise [19], whereas training lowered basal MMP-9 in other populations [20,21], and TIMP responses are reported to rise, fall or remain unchanged depending on the population and modality studied [21,22]. Because mechanical loading is a principal regulator of ECM turnover [12], training modalities that differ in eccentric and impact loading could plausibly differ in their effects on the gelatinase–TIMP system.

Coenzyme Q10 (CoQ10, ubiquinone) is an obligatory electron carrier of the mitochondrial respiratory chain and, in its reduced form (ubiquinol), the only endogenously synthesised lipophilic antioxidant [23]. In well-trained athletes, CoQ10 status may be marginal: college athletes were found to have lower leukocyte CoQ10 than untrained controls, although plasma CoQ10 did not differ between the groups, a pattern attributed to increased mitochondrial demand and oxidative consumption [24], and higher plasma CoQ10 has been associated with better performance and antioxidant capacity [25,26,27]. Because MMP expression is itself redox-sensitive—reactive oxygen species activate pro-MMPs and stimulate MMP-9 transcription [28]—redox status could, in principle, influence MMP/TIMP regulation during intensified training, although direct evidence linking circulating CoQ10 to MMP/TIMP regulation in humans is lacking. The gap addressed here is therefore both practical and physiological: elastic-band and plyometric exercises are routinely added to tennis practice, yet it is unknown whether such supplementary loading, and the type of loading, is associated with changes in circulating markers related to ECM turnover or antioxidant status. To our knowledge, no study has compared these modalities on these markers.

The present study therefore explored whether 8 weeks of elastic-band resistance training or plyometric training, each added to habitual tennis practice, was associated with different changes in serum MMP-2, MMP-9, TIMP-1, TIMP-2 and CoQ10 compared with tennis practice alone in collegiate tennis players. Before data collection, we hypothesised that both modalities would lower basal MMP-9 and the MMP-9/TIMP-1 ratio, as reported after several forms of chronic training [20,21], and that plyometric training would reduce CoQ10 to a greater extent than elastic-band training, because repeated high-velocity eccentric and impact loading induces muscle damage and inflammation [11] that may increase reactive-oxygen-species production and antioxidant utilisation [23,27]. Given the limited sample available, the study was designed as exploratory and hypothesis-generating.

## 2. Materials and Methods

### 2.1. Study Design and Participants

This was an exploratory, three-arm, parallel-group, matched, non-randomised controlled intervention study with pre- and post-intervention measurements. The study was approved by the Non-Interventional Clinical Research Ethics Committee of Van Yüzüncü Yıl University (decision no. 2023/11-09, 10 November 2023), conducted in accordance with the Declaration of Helsinki and supported by the Scientific Research Projects Coordination Unit of Van Yüzüncü Yıl University (project TYL-2023-10777). All participants gave written informed consent. The study and its analysis plan were not prospectively registered, and it is reported according to the TREND statement for non-randomised evaluations; all analyses should therefore be regarded as exploratory.

Twenty-four tennis players (11 women and 13 men; aged 20–25 years, mean 21.7 ± 1.8 years) were recruited from physically active students of the Faculty of Sport Sciences, Van Yüzüncü Yıl University, who had general tennis knowledge and skills and at least 2 years of regular tennis training. These students were studied because they form a relatively homogeneous population training within a common faculty programme, which allowed supplementary training to be added to a shared habitual schedule. The intervention was conducted during the spring semester, between March and May 2024. Inclusion criteria were ≥2 years of regular tennis training, absence of musculoskeletal injury in the previous 6 months, no chronic disease and no use of medication or dietary supplements (including CoQ10 or antioxidants). Participants were allocated to a control group (CON), an elastic-band resistance-training group (RBT) or a plyometric-training group (PLY) using cluster analysis, applied before the start of the study so that participants were divided into groups that were homogeneous for baseline characteristics. Baseline serum markers could not be used for matching because all samples were analysed after the intervention. Allocation was therefore matched rather than randomised, and neither participants nor supervising investigators were blinded to group. Eight participants were initially allocated to each group (Supplementary Figure S1). One participant in the RBT group did not provide a post-intervention blood sample and was excluded from all analyses, leaving 23 participants (CON *n* = 8, RBT *n* = 7, PLY *n* = 8; 10 women and 13 men). The unit of analysis was the individual participant; no data were imputed.

### 2.2. Training Interventions

All groups continued their habitual tennis training throughout the study; habitual training volume and intensity were not recorded individually (e.g., session rating of perceived exertion or duration logs), so equivalence of habitual load between groups could not be verified. RBT and PLY additionally performed supervised training on Mondays, Wednesdays and Fridays for 8 weeks (24 sessions). Each session comprised a 10-min warm-up of jogging and dynamic stretching, a 30–60-min main part and a 5-min cool-down of static stretching [29]. Programmes were designed from the literature and reviewed by three university faculty members with expertise in training science. Volume was progressed identically in both groups: 2 sets × 8 repetitions in weeks 1–2, 3 × 10 in weeks 3–6 and 4 × 12 in weeks 7–8, with 1–2 min rest between sets, for each of eight exercises.

The plyometric programme consisted of four upper-body medicine-ball exercises (plyometric push-ups, squat throws, overhead throws, standing backward high toss) and four lower-body jump exercises (180° turning jumps between cones, split-squat jumps, long jumps with change of direction over 10 m, and squat jumps), all performed with maximal intent. The elastic-band programme consisted of eight Thera-Band (Performance Health, Akron, OH, USA) exercises targeting the trunk, shoulder girdle and hip (penguin crunch, side-plank high pull, side upgrade, standing banded triceps extension, banded side walk, oyster shell, speed repeater leg and bicycle legs). Band resistance was selected individually so that the prescribed repetitions could be completed with correct technique and was increased when this was no longer challenging. Attendance was recorded at every session; all participants in RBT and PLY completed all 24 sessions (100% adherence), and no training-related adverse events occurred. External training volume was matched only by prescription (number of exercises, sets and repetitions); mechanical load, time under tension and internal load (e.g., heart rate or rating of perceived exertion) were not quantified, so the two programmes cannot be assumed to have imposed equal physiological doses. Because CON performed no additional sessions and no sham activity, comparisons of RBT or PLY with CON estimate the combined effect of the added modality and of 24 additional sessions of training exposure, whereas the RBT vs. PLY comparison, with identical session frequency and set–repetition progression, is the contrast most informative about training modality. Dietary intake, including habitual consumption of antioxidant- and CoQ10-rich foods, was neither recorded nor standardised; participants were asked to maintain their usual diet and habitual activity, and use of dietary supplements was an exclusion criterion; absence of supplement use throughout the training period was confirmed at the post-intervention assessment.

### 2.3. Blood Sampling and Biochemical Analyses

Venous blood (5 mL) was drawn from an antecubital vein after an overnight fast, in the morning (08:00–09:00), before the first training session and 1 day (approximately 24 h) after the last supplementary session, into cooled gel serum-separator tubes. The last habitual tennis session also took place 1 day before post-intervention sampling, so that approximately 24 h separated any training from sampling in all three groups. Before each sampling, participants were instructed to abstain from caffeine and alcohol and to refrain from strenuous exercise. Samples were left to clot for 20 min at room temperature, centrifuged at 4000 rpm for 10 min (Centurion Scientific K3 series K241R; Centurion Scientific Ltd., Chichester, UK), and the serum was stored at −80 °C until analysis, which was performed 1 day after the post-intervention sampling (storage time approximately 8 weeks for pre-intervention and 1 day for post-intervention samples). Pre- and post-intervention samples from the same participant were analysed on the same plate in a single batch so that each sample underwent a single freeze–thaw cycle.

Serum MMP-2, MMP-9, TIMP-1, TIMP-2 and CoQ10 were quantified with commercial enzyme-linked immunosorbent assays (Reed Biotech Ltd., Wuhan, China; catalogue nos. RE1838H, RE2796H, RE1094H, RE1845H and RE3180H, respectively) in the Department of Medical Biochemistry, Van Yüzüncü Yıl University, following the manufacturer’s protocol (90-min sample incubation, biotinylated antibody 60 min, HRP conjugate 30 min, TMB substrate 15 min). All five kits are sandwich immunoassays with a biotinylated detection antibody and HRP conjugate. The CoQ10 kit (RE3180H) is designated by the manufacturer as the “Human CoQ10B (Coenzyme Q10 Homolog B) ELISA Kit”, with an antibody pair specific for human CoQ10B; the analyte is referred to as CoQ10 in this article, but the assay does not quantify the ubiquinone or ubiquinol forms of coenzyme Q10 (see Limitations). Absorbance was read at 450 nm on a BioTek 800 microplate reader (BioTek Instruments, Winooski, VT, USA), and concentrations were calculated by the reader software from a standard curve generated on each plate, fitted with a two-parameter logit-log model (four-parameter logistic model for TIMP-1). Coefficients of determination of the standard curves were 0.987 (MMP-2), 0.955 (MMP-9), 0.988 (TIMP-1), 0.983 (TIMP-2) and 0.959 (CoQ10); two of the eight MMP-2 calibrators were excluded from the curve fit by the software. Assay sensitivities were 0.47 ng/mL (MMP-2), 0.10 ng/mL (MMP-9, TIMP-1, TIMP-2) and 0.38 ng/mL (CoQ10); detection ranges were 0.79–50, 0.16–10, 0.16–10, 0.16–10 and 0.63–40 ng/mL, respectively. According to the manufacturer, intra- and inter-assay coefficients of variation (three samples, each measured 20 times) were 2.7–7.9% for all analytes, spike recovery in serum averaged 87–100% and dilution linearity was 81–106% (Supplementary Table S1). Samples were assayed undiluted in single wells, although the manufacturer recommends duplicate measurement, because of the limited number of kits available within the project budget; consequently, within-run imprecision could not be estimated from the study samples, and no study-specific validation (spike recovery, parallelism or comparison with a chromatographic method) was performed. Reported units (ng/mL) and calculations were re-checked against the kit standards and the raw optical densities; because samples were undiluted, no dilution factor was applied. The absolute concentrations of all analytes were lower than values commonly reported for human serum, which may reflect calibrator assignment and antibody specificity of these kits, which are not harmonised with other immunoassays or with reference methods; concentrations should therefore be compared only within this study. MMP-9/TIMP-1 and MMP-2/TIMP-2 mass-concentration ratios are reported as descriptive surrogates; because they are not molar and the assays measure total protein rather than activity, they index relative change rather than stoichiometric inhibition or net proteolytic activity.

### 2.4. Statistical Analysis

The unit of replication was the participant (CON *n* = 8, RBT *n* = 7, PLY *n* = 8). Because the sample size was fixed by the thesis design, no a priori power calculation was performed; instead, a sensitivity analysis showed that with α = 0.05 and 80% power, the study could detect a between-group effect of Cohen’s f ≥ 0.70 for the three-group comparison (equivalent to ηp^2^ ≥ 0.33) and a pairwise standardised difference of d ≥ 1.57. The study was therefore underpowered to detect small-to-moderate effects: non-significant results are inconclusive rather than evidence of no effect, and statistically significant effects are likely to be overestimated. Data are reported as mean ± SD or, where distributions were skewed, as median (interquartile range), together with the number of participants contributing to each estimate.

Distributional assumptions were assessed with the Shapiro–Wilk test on model residuals and Levene’s test (median-centred) for homogeneity of variance. TIMP-1, MMP-9 and both MMP/TIMP ratios were right-skewed and were natural-log transformed before analysis; for these outcomes, effects are reported as back-transformed ratios of geometric means. The primary analysis for each analyte was analysis of covariance (ANCOVA) of the post-intervention value with group as a fixed factor and the baseline value as a covariate. ANCOVA was chosen because, in small two-time-point studies prone to baseline imbalance and regression to the mean, it yields less biased and more precise estimates of between-group differences than comparison of change scores [30,31]. Homogeneity of regression slopes was verified by testing the group × baseline interaction. Baseline-adjusted mean differences between groups (RBT − CON, PLY − CON and RBT − PLY) are reported with 95% confidence intervals (CI), standardised as Hedges’ g using the pooled within-group baseline SD with a small-sample correction (J = 1 − 3/(4df − 1), df = 20), and *p* values for the three pairwise contrasts within each analyte were adjusted with the Holm procedure. Partial η^2^ is reported for the overall group effect, and because five analytes were tested, the corresponding *p* values were additionally controlled for the false-discovery rate (Benjamini–Hochberg q). The two procedures address different families of tests: Holm adjustment controls the family-wise error rate for the three pairwise contrasts within an analyte, whereas the Benjamini–Hochberg procedure controls the false-discovery rate across the five analytes. No single primary outcome was specified before data collection; the five analytes were treated as co-primary exploratory outcomes and the two MMP/TIMP ratios as secondary outcomes. Effect estimates with 95% CIs, rather than *p* values, are the basis of interpretation.

Three sensitivity analyses were performed: (i) ANCOVA with heteroscedasticity-consistent (HC3) standard errors; (ii) a rank-based analysis in which change scores were compared across groups with the Kruskal–Wallis test followed by Holm-adjusted Dunn tests; and (iii) ANCOVA with sex added as a covariate. HC3 *p* values are unadjusted, whereas Dunn tests were Holm-adjusted. A between-group finding was considered robust only if the primary ANCOVA contrast was significant after Holm adjustment and was supported by all three sensitivity analyses; findings that were significant in the primary analysis but not supported by one or more sensitivity analyses were classified as sensitive to analytical assumptions. A 3 (group) × 2 (time) mixed-model ANOVA with participant as the random factor was also fitted to report the time main effect and the group × time interaction. Within-group changes are described as the mean paired difference with its 95% CI, the paired t test (or Wilcoxon signed-rank test when the differences departed from normality) and Hedges’ g_av_, which uses the square root of the mean of the pre- and post-intervention variances as the standardiser, with a small-sample correction (df = *n* − 1) [32]. Exploratory associations among the change scores of the five analytes, including the biologically paired MMP-2/TIMP-2 and MMP-9/TIMP-1, were examined with Spearman’s ρ (10 correlations, Benjamini–Hochberg adjusted). Correlations between baseline values and change scores were not used to assess regression to the mean, because such correlations are affected by mathematical coupling [33]; regression to the mean was instead addressed by baseline adjustment [30,31]. All tests were two-sided with α = 0.05.

Additional exploratory analyses—permutational multivariate analysis of variance (PERMANOVA) of the five-analyte change profile with principal component analysis [34], equivalence testing by two one-sided tests, bootstrap probabilities relative to the smallest worthwhile change and estimation of inter-individual response variability [35,36]—were performed to describe the data. Because of the multiplicity and analytical flexibility they introduce in a sample of this size, they were not used for inference and are reported, with their methods, only in the Supplementary Materials (Tables S2 and S3, Figure S2). Analyses were performed in Python 3.12 with SciPy 1.17, statsmodels 0.14 and pingouin 0.6; the analysis code is available from the corresponding author.

## 3. Results

### 3.1. Participants and Baseline Characteristics

**Figure 1:**
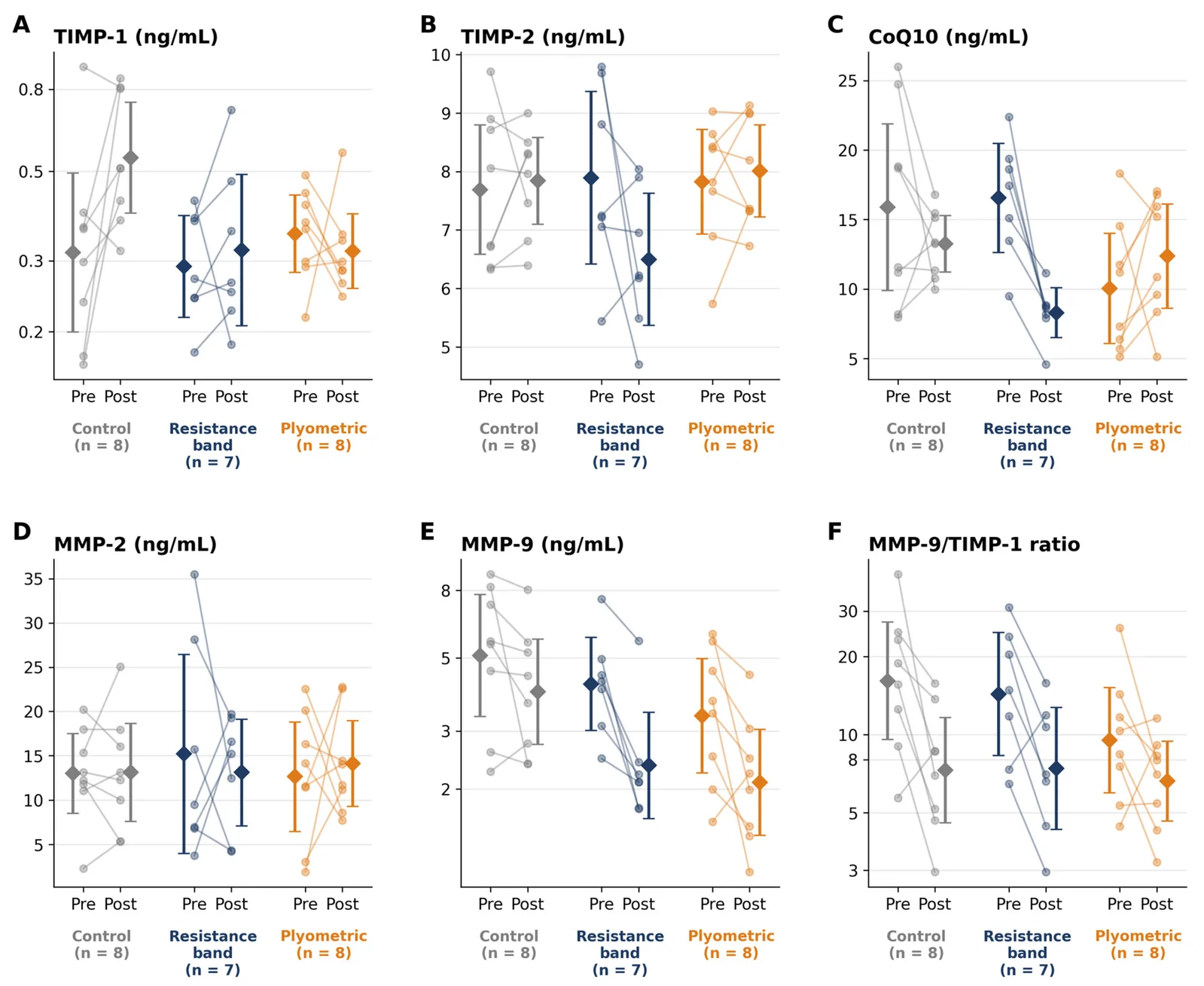
Individual pre- and post-intervention serum concentrations of (**A**) TIMP-1, (**B**) TIMP-2, (**C**) coenzyme Q10, (**D**) MMP-2, (**E**) MMP-9 and (**F**) the MMP-9/TIMP-1 ratio in the control (grey), elastic-band resistance-training (navy) and plyometric-training (orange) groups. Thin lines connect the two samples from each participant; diamonds with whiskers show the group mean and its 95% confidence interval (geometric mean and CI on the log scale for panels A, E and F, which are drawn on logarithmic axes). *n* denotes participants per group (CON 8, RBT 7, PLY 8); each participant contributed one serum sample per time point.

Twenty-three players completed both assessments (Table 1). The sex distribution and baseline TIMP-1, TIMP-2, MMP-2 and MMP-9 did not differ between groups; baseline CoQ10 was lower in PLY than in CON and RBT, although the difference was not statistically significant (*p* = 0.054). ANCOVA assumptions were met for all outcomes except homogeneity of variance for CoQ10 (Levene *p* = 0.017), for which heteroscedasticity-consistent (HC3) standard errors are also reported. Pre- and post-intervention values of all outcomes are given in Table 2 and Figure 1.

### 3.2. Tissue Inhibitors of Metalloproteinases

**Figure 2:**
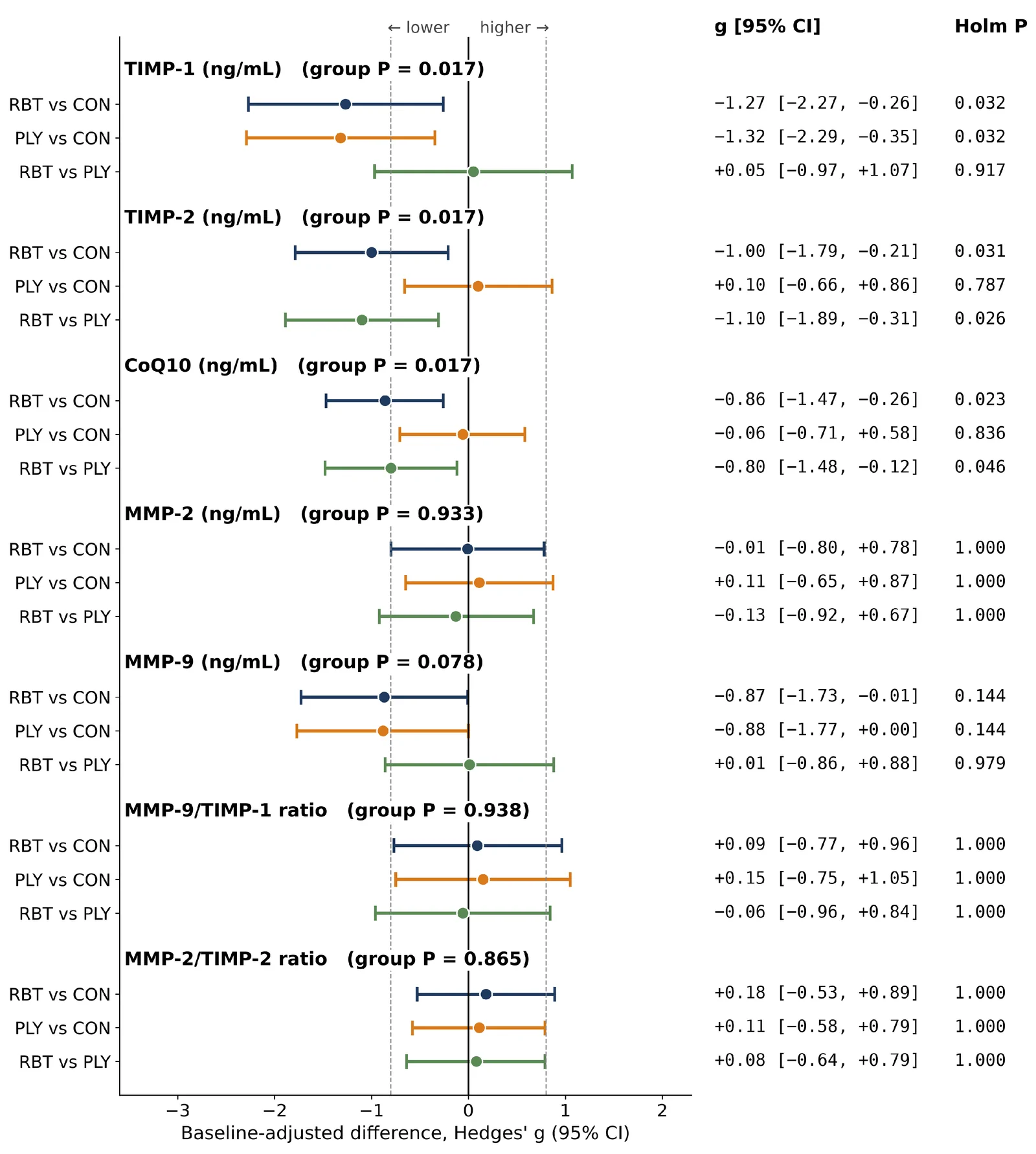
Baseline-adjusted between-group differences after 8 weeks, expressed as Hedges’ g (points) with 95% confidence intervals (horizontal lines), from analysis of covariance of the post-intervention value with the baseline value as covariate (log scale for TIMP-1, MMP-9 and both ratios). Dashed lines mark |g| = 0.8. Numbers at right give g [95% CI] and the Holm-adjusted *p* value for the three pairwise contrasts within each outcome; the overall group-effect *p* value is shown beside each outcome name. Negative values indicate lower adjusted values in the first-named group. CON, control (*n* = 8); RBT, elastic-band resistance training (*n* = 7); PLY, plyometric training (*n* = 8).

**Figure 3:**
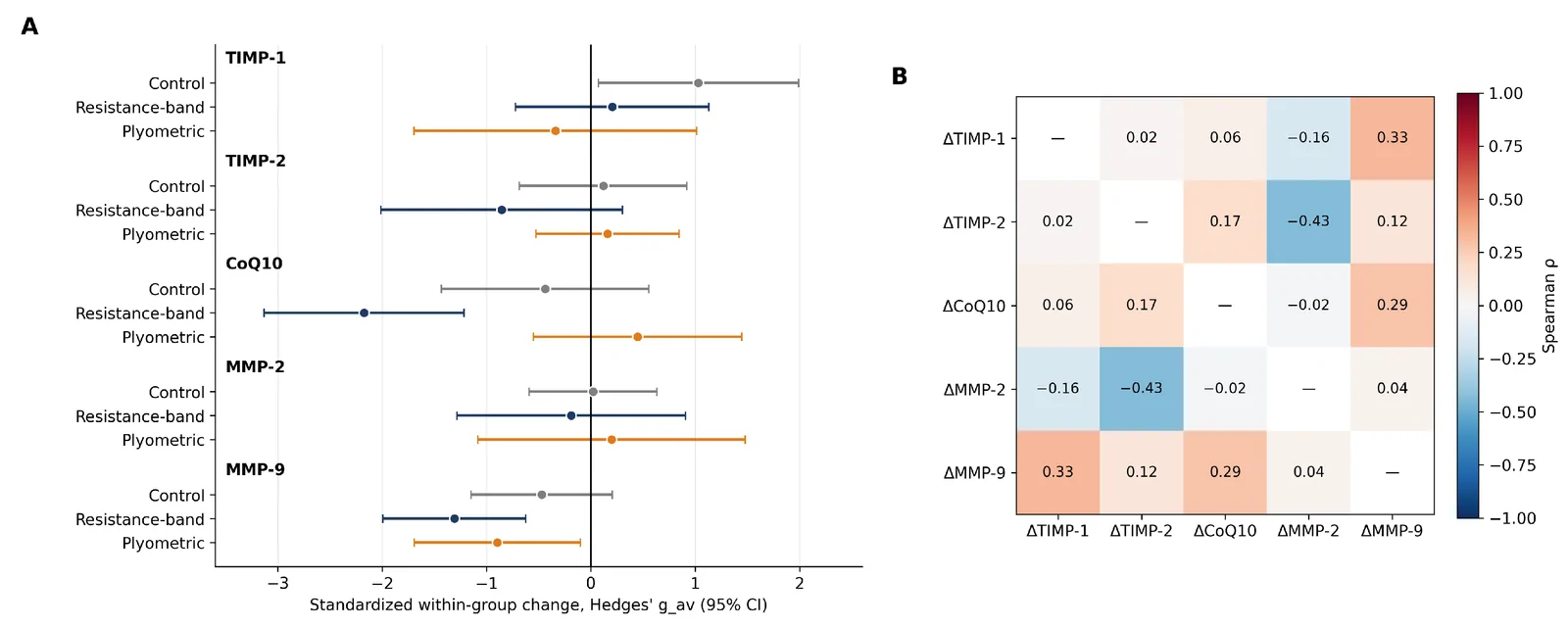
(**A**) Standardised within-group change from baseline (Hedges’ g_av_, points) with 95% confidence intervals for the five analytes in each group (CON *n* = 8, RBT *n* = 7, PLY *n* = 8); the interval is the 95% CI of the mean paired difference divided by the root mean square of the pre- and post-intervention SDs, with small-sample correction. Values for TIMP-1 and MMP-9 are on the natural-log scale. (**B**) Spearman rank correlations among the change scores (Δ = post − pre; log-scale for TIMP-1 and MMP-9) of the five analytes across all 23 participants. No correlation remained significant after Benjamini–Hochberg adjustment of the 10 tests.

Baseline-adjusted TIMP-1 was lower in RBT and PLY than in CON (Holm *p* = 0.032 for both; Table 3, Figure 2) and did not differ between the two training groups. This difference did not reflect a decrease in the training groups: TIMP-1 increased within CON (×1.72, *p* = 0.039) and was unchanged in RBT and PLY (Table 2, Figure 1A, Figure 3A), and the group × time interaction was not significant (*p* = 0.062). The PLY vs. CON difference was supported by all sensitivity analyses, whereas the RBT vs. CON difference was robust to sex adjustment but was not confirmed by the HC3 (*p* = 0.054) or rank-based (*p* = 0.480) analyses (Table 3 and Table 4).

Baseline-adjusted TIMP-2 was lower in RBT than in CON (Holm *p* = 0.031) and PLY (Holm *p* = 0.026), whereas PLY and CON did not differ (Table 3, Figure 2). The within-group decrease in RBT was not significant (*p* = 0.12; Table 2, Figure 1B), the group × time interaction was not significant (*p* = 0.081), and the rank-based analysis of unadjusted change scores did not confirm the group difference (Kruskal–Wallis *p* = 0.24; Dunn–Holm *p* = 0.34 for RBT vs. CON and 0.36 for RBT vs. PLY), although the HC3 (Table 3) and sex-adjusted analyses did. The TIMP-2 difference is therefore classified as sensitive to analytical assumptions (Table 4).

### 3.3. Coenzyme Q10

CoQ10 decreased in RBT (*p* = 0.001, g_av_ = −2.17) but not in CON or PLY (Table 2, Figure 1C), yielding a group × time interaction (*p* = 0.010). Baseline-adjusted CoQ10 was lower in RBT than in CON (Holm *p* = 0.023; HC3 *p* = 0.001; Dunn–Holm *p* = 0.262) and PLY (Holm *p* = 0.046; HC3 *p* = 0.064; Dunn–Holm *p* = 0.010), whereas PLY and CON did not differ (Table 3). After adjustment for sex, the RBT vs. CON difference persisted (Holm *p* = 0.031), whereas the RBT vs. PLY difference was attenuated (Holm *p* = 0.062). Each RBT contrast was therefore supported by the within-group change, the interaction and some, but not all, sensitivity analyses (Table 4). Because RBT had the highest and PLY the lowest baseline CoQ10, part of the RBT decline may reflect regression to the mean, which ANCOVA corrects only partially when allocation is not randomised [31].

### 3.4. Matrix Metalloproteinases and MMP/TIMP Balance

MMP-9 decreased over time in all three groups (time *p* < 0.001; within-group *p* = 0.078, 0.003 and 0.032 in CON, RBT and PLY, respectively; Table 2, Figure 1E) without a group × time interaction (*p* = 0.36). Baseline-adjusted MMP-9 did not differ between groups after correction (group *p* = 0.078; Holm *p* = 0.14 for both training groups vs. CON; Table 3). MMP-2 was unchanged over time (Figure 1D) and did not differ between groups (Table 3); the confidence intervals were wide (e.g., RBT vs. CON g −0.01, 95% CI −0.80 to 0.78), so moderate differences cannot be excluded. MMP-2 also showed a low pre–post correlation across participants (r = 0.05), with several individual concentrations changing by up to 12-fold; the raw optical densities and calculations for these samples were re-checked, but analytical variability cannot be excluded. The MMP-9/TIMP-1 ratio decreased over time (time *p* < 0.001; within-group *p* = 0.012, 0.031 and 0.140 in CON, RBT and PLY; Figure 1F), and the MMP-2/TIMP-2 ratio was unchanged; neither ratio differed between groups (Table 3).

### 3.5. Associations Among Change Scores and Sex Differences

Changes in the five analytes, including the biologically paired MMP-2/TIMP-2 and MMP-9/TIMP-1 and the pairs involving CoQ10, were largely independent (Figure 3B); the inverse association between ΔTIMP-2 and ΔMMP-2 (ρ = −0.43, *p* = 0.039) did not survive false-discovery-rate correction (q = 0.39). Change scores did not differ between women and men for any analyte (*p* = 0.10–1.0). No performance, body-composition or training-load data were available to relate to the biochemical changes. The exploratory multivariate, equivalence, bootstrap and individual-response analyses are reported in Supplementary Tables S2 and S3 and Figure S2; they did not alter the interpretation of the univariate findings.

## 4. Discussion

In this exploratory, non-randomised study of collegiate tennis players, adding 8 weeks of elastic-band resistance training to habitual tennis practice was associated with lower baseline-adjusted serum CoQ10 than tennis practice alone or tennis practice plus plyometric training. A similar difference in TIMP-2 was not supported by the rank-based sensitivity analysis and is regarded as preliminary. The lower adjusted TIMP-1 in both training groups reflected an increase in the control group rather than a decrease with training, and MMP-9 decreased over time in all groups, including controls, so neither modality produced a demonstrable training-specific change in circulating gelatinases; MMP-2 did not change. Neither a priori hypothesis was supported. Because the study could detect only very large effects and no tissue-level or oxidative-stress measurements were made, the interpretations below are hypotheses generated by, rather than confirmed by, these data.

The CoQ10 result was the most consistent between-group difference: it was accompanied by a significant within-group decline and group × time interaction and was supported by some, though not all, sensitivity analyses (Table 4). Its biological meaning is nevertheless uncertain. Importantly, the assay is specified by the manufacturer for CoQ10B (coenzyme Q10 homolog B), a mitochondrial CoQ-binding protein, rather than for coenzyme Q10 itself, so the serum values cannot be interpreted directly as circulating coenzyme Q10 or antioxidant status. To the extent that the measurement reflects coenzyme Q10 status, circulating CoQ10 reflects hepatic synthesis, dietary intake and lipoprotein transport as well as tissue uptake and utilisation [23]; because it is carried mainly in low-density lipoproteins, plasma concentrations are usually interpreted relative to cholesterol [37], which was not measured here. A lower serum value therefore does not by itself indicate increased oxidative stress or depleted antioxidant reserves, and oxidative-stress markers were not measured. Possible explanations include increased utilisation or redistribution into skeletal muscle, where CoQ10 content may rise with training [38]; changes in lipoprotein metabolism; uncontrolled dietary variation; regression to the mean, given that RBT had the highest baseline values; or analytical factors related to an immunoassay that targets CoQ10B and has not been compared with chromatographic measurement of coenzyme Q10. Lower circulating CoQ10 has been reported after weeks of training in animals [39], and athletes may show lower cellular, but not plasma, CoQ10 than untrained individuals [24]; supplementation studies suggest that CoQ10 status may influence recovery and performance [26,27,40], but whether a change of the magnitude observed here has any functional consequence is unknown. Contrary to our hypothesis, plyometric training, despite its greater eccentric and impact loading, was not associated with lower CoQ10 (adjusted g −0.06, 95% CI −0.71 to 0.58), although the interval does not exclude a moderate effect. Why a moderate-load, continuous-tension band programme would be associated with lower serum CoQ10 when a plyometric programme with identical volume progression was not cannot be resolved with these data; unquantified differences in metabolic demand or time under tension between the programmes are possibilities.

The TIMP-2 difference after RBT was not accompanied by a significant within-group change or group × time interaction and was not supported by the rank-based analysis, which is less influenced by individual extreme values in small samples; it should therefore be regarded as a preliminary observation. Training effects on circulating TIMP-2 reported in the literature are inconsistent in direction, with decreases in some animal and clinical models [22,41,42] and increases in others [21,43], and because MMP activity and tissue markers were not measured, the observed difference cannot be linked to matrix remodelling. The TIMP-1 result illustrates the value of a concurrent control group: an unadjusted post-intervention comparison would have suggested a training-induced decrease, whereas the data show an unexplained 72% increase in controls with stable values in the training groups. TIMP-1 responds to inflammatory and other stimuli [15], so this rise could reflect changes in the controls’ habitual training or other exposures, chance variation in a group of eight or measurement uncertainty, given that TIMP-1 concentrations were close to the lower limit of the assay range. The TIMP-1 difference should therefore not be interpreted as exercise-induced suppression of TIMP-1.

MMP-9 and the MMP-9/TIMP-1 ratio decreased over the study in all groups, including controls, without a group × time interaction. A shared decline most plausibly reflects factors common to all participants—such as a change in the phase of the tennis season or in habitual activity, differences between the two sampling occasions or regression to the mean—rather than an effect of the supplementary programmes. Reductions in basal MMP-9 after chronic training have been reported in clinical populations [20,21,44] and aged animals [45], whereas 8 weeks of resistance training did not change resting serum MMP-9 in healthy young adults [19], consistent with the absence of a training-specific effect here; the confidence intervals, however, do not exclude an additional training-related reduction of moderate size. MMP-2 did not change in any group. This is biologically plausible, because MMP-2 is constitutively expressed and regulated mainly at the level of activation by MT1-MMP and TIMP-2 [13,14], and increases in muscle or circulating MMP-2 have generally followed unaccustomed eccentric or electrically stimulated contractions causing overt tissue disruption [46,47] rather than progressive, familiarised training. The apparently greater responsiveness of TIMP-1, TIMP-2 and CoQ10 than of the gelatinases should nevertheless be interpreted cautiously, because MMP-2 showed large within-participant variability that may be partly analytical.

Two features of the design limit inference about tissue adaptation. First, serum MMPs and TIMPs are released by many tissues and cells, including leukocytes, platelets, endothelium and liver, and the assays measured total immunoreactive protein rather than enzymatic activity; changes in circulating concentrations therefore cannot be taken as evidence of corresponding changes in muscle, tendon or connective-tissue remodelling, and the MMP/TIMP ratios are descriptive surrogates rather than measures of net proteolytic balance. Second, post-intervention blood was collected approximately 24 h after the final supplementary session. Plyometric exercise induces muscle damage and acute inflammatory responses that peak 24–48 h after exercise [11], and MMP responses to a single bout of exercise can persist for hours to days [17,18]. Post-intervention values may therefore combine chronic adaptation with residual responses to the final sessions; because both training groups were sampled at the same interval, this would affect comparisons with CON more than the RBT vs. PLY comparison. Sampling 48–72 h after the final session would better characterise resting adaptations in future studies.

For coaches and practitioners, the present findings do not justify changes to training prescription. No performance, recovery or injury outcomes were measured, the physiological significance of the observed biomarker differences is uncertain, and the CoQ10 assay targets CoQ10B rather than coenzyme Q10 itself. At most, the data suggest that circulating CoQ10, measured chromatographically and alongside oxidative-stress markers, training load and performance, may merit attention in adequately powered studies of supplementary resistance training in tennis players.

Strengths include a concurrent control group that continued sport-specific training, supervised interventions with matched volume progression and complete adherence, single-batch analysis of paired samples and reporting of effect sizes with confidence intervals and sensitivity analyses. The limitations are substantial. First, allocation was matched but not randomised, so residual confounding by unmeasured characteristics cannot be excluded; baseline adjustment does not correct for such confounding, and with unequal baseline values, ANCOVA may itself be biased (Lord’s paradox). Second, the sample was small: only very large effects (f ≥ 0.70; pairwise d ≥ 1.57) were detectable with 80% power, so small-to-moderate effects may have been missed, significant effects are probably overestimated, and non-significant results, including those for plyometric training and MMP-2, are inconclusive. Third, CON received no additional sessions, so comparisons with CON confound modality with additional training exposure, and habitual tennis load, training intensity, diet, sleep and menstrual-cycle phase were neither quantified nor controlled. Fourth, no primary outcome was prespecified, five analytes were tested, and the study was not registered; the findings are hypothesis-generating. Fifth, the assays have important analytical limitations: all analytes were measured by commercial ELISA in single wells without study-specific validation; absolute concentrations were lower than values commonly reported for human serum, so results can be interpreted only as relative differences within this study; low pre–post correlations for several analytes (r = −0.01 to 0.23 for TIMP-1, TIMP-2, CoQ10 and MMP-2, compared with 0.71 for MMP-9) and concentrations near the limits of the assay ranges suggest considerable analytical variability; and the CoQ10 assay is specified by the manufacturer for CoQ10B (coenzyme Q10 homolog B) rather than for coenzyme Q10 itself, so the CoQ10 results cannot be equated with ubiquinone/ubiquinol concentrations measured by high-performance liquid chromatography or mass spectrometry, the reference methods, and were not normalised to cholesterol [37]. Sixth, oxidative-stress markers, antioxidant capacity, tissue CoQ10, MMP activity and tissue-level outcomes were not measured, and a single sample at each time point cannot separate day-to-day biological variation from training effects. Finally, the sex distribution was unbalanced in the small RBT group (four women, three men), although sex adjustment did not alter the TIMP findings or the CoQ10 difference between RBT and CON (Holm *p* = 0.031), whereas the CoQ10 difference between RBT and PLY was attenuated (Holm *p* = 0.062).

## 5. Conclusions

In conclusion, in this small, exploratory, non-randomised study of collegiate tennis players, 8 weeks of elastic-band resistance training added to habitual practice was associated with lower baseline-adjusted serum CoQ10 than tennis practice alone or plyometric training; a similar difference in TIMP-2 was not supported by all sensitivity analyses and should be considered preliminary. The lower TIMP-1 in both training groups reflected a rise in the control group, not a training-induced decrease, and MMP-9 declined in all groups. Plyometric training was not associated with changes beyond those observed in all groups, but because the study was powered only for very large effects, this should not be interpreted as evidence of no effect. Because the CoQ10 assay targets CoQ10B rather than the coenzyme itself, whether the lower serum CoQ10 reflects altered antioxidant status, redistribution or analytical factors cannot be determined from these data. Moreover, because the CoQ10 differences were significant in the parametric (ANCOVA) analysis but were only partly supported by the non-parametric (Kruskal–Wallis/Dunn) analysis, the CoQ10 findings should be regarded as preliminary, and future studies with larger, randomised cohorts are necessary to confirm them. These findings are hypothesis-generating and require confirmation in adequately powered randomised trials that include an exercise-matched control condition, quantify habitual and supplementary training load, measure CoQ10 chromatographically and assess oxidative-stress and tissue-level outcomes.

## Figures and Tables

**Table 1:** Participant characteristics and baseline serum concentrations by group. Values are median (interquartile range) unless stated; *n* = number of participants.

Characteristic	Control (*n* = 8)	Resistance-Band (*n* = 7)	Plyometric (*n* = 8)	*p* Value
Women/men, *n*	3/5	4/3	3/5	0.76 ^a^
Age, years	20–25	20–25	20–25	—
TIMP-1, ng/mL	0.33 (0.22–0.37)	0.27 (0.24–0.38)	0.37 (0.30–0.42)	0.53 ^b^
TIMP-2, ng/mL	7.40 (6.63–8.76)	7.24 (7.14–9.25)	8.11 (7.47–8.48)	0.88 ^b^
CoQ10, ng/mL	15.13 (10.44–20.30)	17.43 (14.30–19.01)	9.26 (6.21–12.46)	0.054 ^b^
MMP-2, ng/mL	12.67 (11.58–15.99)	9.49 (6.90–21.94)	12.85 (9.34–17.28)	0.98 ^b^
MMP-9, ng/mL	5.56 (4.07–7.49)	4.25 (3.56–4.70)	3.54 (2.39–4.83)	0.23 ^b^
MMP-9/TIMP-1 ratio	17.24 (11.67–23.65)	14.85 (9.56–22.13)	9.38 (7.00–12.34)	0.21 ^b^
MMP-2/TIMP-2 ratio	1.78 (1.39–2.01)	0.97 (0.97–3.04)	1.90 (1.12–2.39)	0.98 ^b^

^a^ Fisher–Freeman–Halton exact test. ^b^ Kruskal–Wallis test across the three groups. CON, control; RBT, elastic-band resistance training; PLY, plyometric training; MMP, matrix metalloproteinase; TIMP, tissue inhibitor of metalloproteinases; CoQ10, coenzyme Q10.

**Table 2:** Pre- and post-intervention serum concentrations, within-group change and mixed-model ANOVA results for each outcome.

Outcome	Group	Pre, Mean ± SD	Post, Mean ± SD	Change (95% CI) ^a^	*g*_av_	*p* (Within) ^b^	Time *p*; Group × Time *p* (ηp^2^)
TIMP-1 (ng/mL)	Control (*n* = 8)	0.36 ± 0.24	0.58 ± 0.21	×1.72 (1.04–2.85)	1.03	0.039	0.108; 0.062 (0.24)
	RBT (*n* = 7)	0.30 ± 0.09	0.35 ± 0.18	×1.10 (0.72–1.68)	0.20	0.609	
	PLY (*n* = 8)	0.36 ± 0.09	0.33 ± 0.10	×0.91 (0.61–1.34)	−0.34	0.383 ^c^	
TIMP-2 (ng/mL)	Control (*n* = 8)	7.69 ± 1.32	7.84 ± 0.89	+0.15 (−0.86 to 1.17)	0.12	0.734	0.317; 0.081 (0.22)
	RBT (*n* = 7)	7.89 ± 1.59	6.50 ± 1.22	−1.40 (−3.29 to 0.50)	−0.85	0.121	
	PLY (*n* = 8)	7.83 ± 1.07	8.01 ± 0.94	+0.18 (−0.60 to 0.96)	0.16	0.597	
CoQ10 (ng/mL)	Control (*n* = 8)	15.89 ± 7.17	13.26 ± 2.43	−2.64 (−8.62 to 3.35)	−0.44	0.332	0.049; 0.010 (0.37)
	RBT (*n* = 7)	16.56 ± 4.25	8.29 ± 1.94	−8.26 (−11.91 to −4.62)	−2.17	0.001	
	PLY (*n* = 8)	10.05 ± 4.73	12.37 ± 4.48	+2.32 (−2.86 to 7.49)	0.45	0.324	
MMP-2 (ng/mL)	Control (*n* = 8)	13.00 ± 5.39	13.13 ± 6.61	+0.14 (−4.01 to 4.29)	0.02	0.939	0.972; 0.799 (0.02)
	RBT (*n* = 7)	15.20 ± 12.13	13.10 ± 6.52	−2.10 (−14.38 to 10.17)	−0.19	0.690	
	PLY (*n* = 8)	12.63 ± 7.37	14.11 ± 5.77	+1.48 (−8.04 to 11.00)	0.20	0.724	
MMP-9 (ng/mL)	Control (*n* = 8)	5.62 ± 2.45	4.30 ± 1.95	×0.78 (0.54–1.12)	−0.47	0.078 ^c^	<0.001; 0.357 (0.10)
	RBT (*n* = 7)	4.40 ± 1.62	2.57 ± 1.37	×0.57 (0.42–0.76)	−1.31	0.003	
	PLY (*n* = 8)	3.66 ± 1.61	2.29 ± 1.07	×0.63 (0.42–0.95)	−0.90	0.032	
MMP-9/TIMP-1	Control (*n* = 8)	18.95 ± 11.31	8.31 ± 4.45	×0.45 (0.26–0.79)	−1.19	0.012	<0.001; 0.372 (0.09)
	RBT (*n* = 7)	16.53 ± 9.03	8.49 ± 4.53	×0.52 (0.32–0.84)	−0.98	0.031 ^c^	
	PLY (*n* = 8)	10.99 ± 6.83	7.11 ± 2.72	×0.70 (0.42–1.16)	−0.65	0.140	
MMP-2/TIMP-2	Control (*n* = 8)	1.70 ± 0.74	1.70 ± 0.89	×1.00 (0.62–1.61)	0.00	0.998	0.516; 0.872 (0.01)
	RBT (*n* = 7)	2.04 ± 1.66	2.11 ± 1.19	×1.18 (0.35–3.99)	0.17	0.756	
	PLY (*n* = 8)	1.66 ± 0.96	1.75 ± 0.65	×1.32 (0.49–3.51)	0.34	0.526	

Values are mean ± SD of *n* participants per group. ^a^ Mean paired difference (post − pre) with 95% CI; for log-analysed outcomes (TIMP-1, MMP-9 and both ratios), the change is given as the ratio of geometric means (post/pre, ×) with 95% CI. ^b^ Paired t test on the analysis scale. ^c^ Wilcoxon signed-rank test used because paired differences departed from normality (Shapiro–Wilk *p* < 0.05). g_av_, Hedges’ g standardised by the root mean square of the pre- and post-intervention SDs, with small-sample correction. Time *p* and Group × time *p* are from a 3 (group) × 2 (time) mixed-model ANOVA with *F*(1,20) and *F*(2,20), respectively; ηp^2^, partial eta squared for the interaction. Abbreviations as in Table 1.

**Table 3:** Primary analysis: baseline-adjusted between-group differences after 8 weeks (analysis of covariance with the baseline value as covariate), with sensitivity analyses.

Outcome	Group Effect F(2,19); *p*; *ηp*^2^; *q* ^a^	Contrast	Adjusted Difference (95% CI) ^b^	Hedges’ *g* (95% CI)	*p*; Holm *p*	HC3 *p* ^c^	Dunn–Holm *p* ^d^
TIMP-1 (ng/mL)	5.13; 0.017; 0.35; 0.028	RBT vs. CON	×0.59 (0.39–0.90)	−1.27 (−2.27 to −0.26)	0.016; 0.032	0.054	0.480
		PLY vs. CON	×0.58 (0.39–0.87)	−1.32 (−2.29 to −0.35)	0.011; 0.032	0.017	0.045
		RBT vs. PLY	×1.02 (0.67–1.56)	0.05 (−0.97 to 1.07)	0.917; 0.917	0.934	0.480
TIMP-2 (ng/mL)	5.13; 0.017; 0.35; 0.028	RBT vs. CON	−1.39 (−2.48 to −0.30)	−1.00 (−1.79 to −0.21)	0.016; 0.031	0.040	0.337
		PLY vs. CON	+0.14 (−0.92 to 1.19)	0.10 (−0.66 to 0.86)	0.787; 0.787	0.764	0.796
		RBT vs. PLY	−1.53 (−2.62 to −0.44)	−1.10 (−1.89 to −0.31)	0.009; 0.026	0.026	0.362
CoQ10 (ng/mL)	5.10; 0.017; 0.35; 0.028	RBT vs. CON	−5.02 (−8.54 to −1.50)	−0.86 (−1.47 to −0.26)	0.008; 0.023	0.001	0.262
		PLY vs. CON	−0.38 (−4.12 to 3.37)	−0.06 (−0.71 to 0.58)	0.836; 0.836	0.867	0.262
		RBT vs. PLY	−4.65 (−8.58 to −0.71)	−0.80 (−1.48 to −0.12)	0.023; 0.046	0.064	0.010
MMP-2 (ng/mL)	0.07; 0.933; 0.01; 0.933	RBT vs. CON	−0.12 (−7.16 to 6.91)	−0.01 (−0.80 to 0.78)	0.971; 1.000	0.974	1.000
		PLY vs. CON	+1.00 (−5.76 to 7.75)	0.11 (−0.65 to 0.87)	0.761; 1.000	0.774	1.000
		RBT vs. PLY	−1.12 (−8.17 to 5.93)	−0.13 (−0.92 to 0.67)	0.743; 1.000	0.763	1.000
MMP-9 (ng/mL)	2.92; 0.078; 0.24; 0.098	RBT vs. CON	×0.66 (0.44–1.00)	−0.87 (−1.73 to −0.01)	0.048; 0.144	0.052	0.192
		PLY vs. CON	×0.66 (0.43–1.00)	−0.88 (−1.77 to 0.00)	0.051; 0.144	0.062	0.394
		RBT vs. PLY	×1.01 (0.67–1.52)	0.01 (−0.86 to 0.88)	0.979; 0.979	0.981	0.545
MMP-9/TIMP-1	0.06; 0.938; 0.01; 0.938	RBT vs. CON	×1.06 (0.62–1.81)	0.09 (−0.77 to 0.96)	0.823; 1.000	0.849	0.730
		PLY vs. CON	×1.10 (0.63–1.91)	0.15 (−0.75 to 1.05)	0.729; 1.000	0.756	0.392
		RBT vs. PLY	×0.97 (0.55–1.68)	−0.06 (−0.96 to 0.84)	0.896; 1.000	0.908	0.730
MMP-2/TIMP-2	0.15; 0.865; 0.02; 0.938	RBT vs. CON	×1.17 (0.63–2.17)	0.18 (−0.53 to 0.89)	0.597; 1.000	0.680	1.000
		PLY vs. CON	×1.10 (0.60–2.00)	0.11 (−0.58 to 0.79)	0.753; 1.000	0.743	1.000
		RBT vs. PLY	×1.07 (0.58–1.99)	0.08 (−0.64 to 0.79)	0.823; 1.000	0.841	1.000

^a^ Overall group effect from ANCOVA (df = 2, 19); q, Benjamini–Hochberg false-discovery-rate-adjusted *p* across the five primary analytes (ratios adjusted separately as secondary outcomes). ^b^ Adjusted mean difference between the first- and second-named group (ng/mL) with 95% CI; for TIMP-1, MMP-9 and both ratios (analysed on the log scale), the difference is expressed as the ratio of adjusted geometric means (×). Hedges’ g is the adjusted difference standardised by the pooled within-group baseline SD on the analysis scale, with small-sample correction (df = 20); its CI is the CI of the adjusted difference on the same scale. Holm *p*, *p* adjusted for the three pairwise contrasts within each outcome. ^c^ *p* from the same ANCOVA with heteroscedasticity-consistent (HC3) standard errors. HC3 *p* values are unadjusted. ^d^ Holm-adjusted Dunn test comparing unadjusted change scores after a Kruskal–Wallis test. Abbreviations as in Table 1. The group-effect *p* values for TIMP-1, TIMP-2 and CoQ10 coincide when rounded to three decimals because their F statistics are nearly identical (5.133, 5.131 and 5.100), and their q values are identical because the Benjamini–Hochberg step-up procedure assigns tied adjusted values to these three analytes.

**Table 4:** Robustness of the between-group findings across the primary and sensitivity analyses.

Finding	Primary ANCOVA, Holm *p*	HC3 *p*	Sex-Adjusted ANCOVA, Holm *p*	Rank-Based, Dunn–Holm *p*	Within-Group Change; G×T *p*	Interpretation
CoQ10: RBT < CON	0.023	0.001	0.031	0.262	RBT −50% (*p* = 0.001); G×T 0.010	Most consistent finding; not confirmed by rank-based analysis
CoQ10: RBT < PLY	0.046	0.064	0.062	0.010	As above	Not confirmed by HC3 or sex-adjusted analysis
TIMP-1: PLY < CON	0.032	0.017	0.028	0.045	CON ×1.72 (*p* = 0.039); G×T 0.062	Consistent, but due to a rise in CON, not a fall with training
TIMP-1: RBT < CON	0.032	0.054	0.028	0.480	As above	Sensitive to assumptions; due to a rise in CON
TIMP-2: RBT < CON	0.031	0.040	0.017	0.337	RBT *p* = 0.12; G×T 0.081	Sensitive to assumptions; preliminary
TIMP-2: RBT < PLY	0.026	0.026	0.013	0.362	As above	Sensitive to assumptions; preliminary
MMP-9: decrease over time	Group *p* = 0.078	—	—	—	Time *p* < 0.001; G×T 0.357	Common to all groups; no training-specific effect
MMP-2 and MMP/TIMP ratios	Group *p* ≥ 0.865	—	—	—	G×T *p* ≥ 0.372	No difference detected; inconclusive

Primary ANCOVA *p* values are Holm-adjusted for the three contrasts within each analyte; HC3 *p* values are unadjusted; Dunn–Holm *p* values are from the Kruskal–Wallis/Dunn analysis of unadjusted change scores. Sex-adjusted ANCOVA *p* values are Holm-adjusted for the three contrasts within each analyte. G×T, group × time interaction from the 3 × 2 mixed-model ANOVA. A finding was classified as robust only if supported by the primary and all sensitivity analyses; no between-group finding met this criterion. Abbreviations as in Table 1.

## Data Availability

The de-identified individual-participant dataset and analysis code are available from the corresponding author on reasonable request.

## Supplementary Materials

The following supporting information can be downloaded at: https://www.mdpi.com/article/10.3390/metabo16100801/s1, Figure S1: Flow of participants through the study; Figure S2: Principal component analysis of change scores and inter-individual response variability; Table S1: Characteristics and manufacturer-reported performance of the enzyme-linked immunosorbent assays; Table S2: Exploratory bootstrap, equivalence and individual-response analyses; Table S3: Permutational multivariate analysis of variance of the five-analyte change profile.

## Abbreviations

ANCOVA, analysis of covariance; ANOVA, analysis of variance; CI, confidence interval; CON, control group; CoQ10, coenzyme Q10; ECM, extracellular matrix; ELISA, enzyme-linked immunosorbent assay; HC3, heteroscedasticity-consistent standard errors (type 3); HRP, horseradish peroxidase; MMP, matrix metalloproteinase; MT1-MMP, membrane-type 1 matrix metalloproteinase; PCA, principal component analysis; PERMANOVA, permutational multivariate analysis of variance; PLY, plyometric-training group; RBT, elastic-band resistance-training group; SD, standard deviation; SDIR, standard deviation of individual responses; SWC, smallest worthwhile change; TMB, 3,3′,5,5′-tetramethylbenzidine; TIMP, tissue inhibitor of metalloproteinases; TOST, two one-sided tests.
