# Supplementary material for: Effects of Elastic-Band Resistance and Plyometric Training on Serum MMP-2, MMP-9, TIMPs and Coenzyme Q10 in Collegiate Tennis Players: An Exploratory, Non-Randomised Three-Arm Controlled Study
Source: Metabolites. 2026 Oct 21;16(10):801. doi: 10.3390/metabo16100801 (PMC13707224; doi:10.3390/metabo16100801)
Supplement: Supplementary file 1 [file metabolites-16-00801-s001.zip › metabolites-4628370-supplementary.pdf]

Supplementary Materials

Effects of Elastic-Band Resistance and Plyometric Training on Serum MMP-2, MMP-9, TIMPs and Coenzyme Q10 in Collegiate Tennis Players: An Exploratory, Non-Randomised Three-Arm Controlled Study

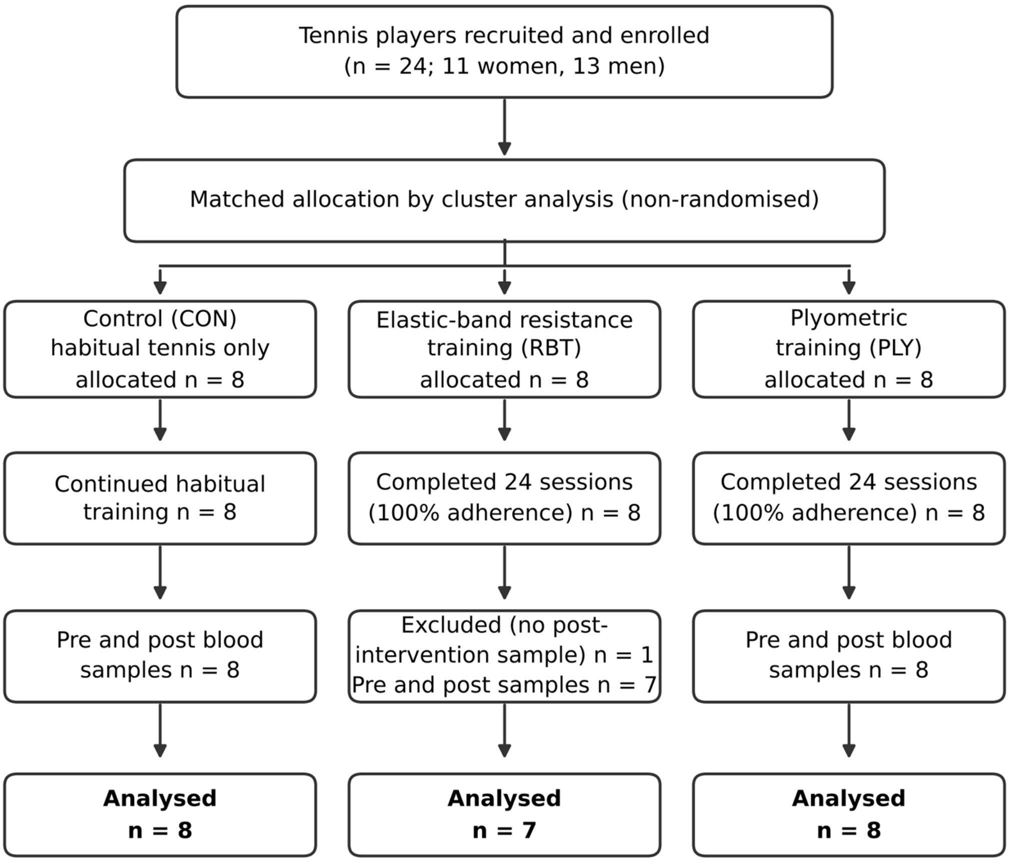

Figure S1. Flow of participants through the study. Allocation was matched by cluster analysis and was not randomised. CON, control; RBT, elastic-band resistance training; PLY, plyometric training.

Table S1. Characteristics and manufacturer-reported performance of the enzyme-linked immunosorbent assays (Reed Biotech Ltd., Wuhan, China).

Analyte	Catalogue no.[a]	Sensitivity (ng/mL)	Detection range (ng/mL)	Intra-assay CV (%)[b]	Inter-assay CV (%)[b]	Recovery in serum, mean (range) %	Dilution linearity, mean %[c]
MMP-2	RE1838H	0.47	0.79–50	2.9–5.7	4.0–7.9	89 (83–95)	87–97
MMP-9	RE2796H	0.10	0.16–10	2.7–5.7	3.8–7.4	100 (84–106)	81–106
TIMP-1	RE1094H	0.10	0.16–10	2.7–7.1	3.9–6.7	87 (80–95)	91–104
TIMP-2	RE1845H	0.10	0.16–10	2.7–7.2	3.0–7.0	100 (96–104)	86–96
CoQ10	RE3180H	0.38	0.63–40	2.9–7.3	2.9–7.4	88 (82–95)	95–100

[a] All kits are sandwich ELISAs read at 450 nm. The CoQ10 kit (RE3180H) is designated by the manufacturer as the "Human CoQ10B (Coenzyme Q10 Homolog B) ELISA Kit". [b] Manufacturer data: three samples (low, medium and high concentration), each measured 20 times; values are the range across the three samples. [c] Range of mean recoveries across 1:2, 1:4, 1:8 and 1:16 dilutions of spiked samples. CV, coefficient of variation.

Supplementary Methods: exploratory, hypothesis-generating analyses

These analyses were not used for confirmatory inference. (i) To describe whether training altered the biochemical profile as a whole, the five change scores (analysis scale) were standardised and compared across

groups by permutational multivariate analysis of variance (PERMANOVA; Euclidean distance, 9,999 permutations, pseudo-F and R^2 [28]), both on raw change scores and on change scores residualised on their baseline values, with pairwise PERMANOVA (4,999 permutations) and principal-component analysis for visualisation. (ii) For non-significant contrasts, equivalence was tested with two one-sided tests (TOST) on the ANCOVA-adjusted difference and its standard error (t distribution, 19 df) using symmetric bounds of ±0.8 pooled within-group baseline SD, and the smallest bound at which equivalence could be declared ($P < 0.05$) is reported. Because ±0.8 SD corresponds to a large standardised effect that is not biologically justified for these analytes, equivalence at this bound excludes only very large differences and is not interpreted as evidence of equivalence or of the absence of a meaningful difference. (iii) The probability that each adjusted difference exceeded the smallest worthwhile change (SWC, 0.2 × pooled within-group baseline SD) in either direction was estimated by stratified non-parametric bootstrap of the ANCOVA (4,000 resamples); these probabilities are descriptive. (iv) Inter-individual variability in the training response was estimated as the SD of individual responses, $SDIR = \sqrt{(SD^2\Delta,\text{training} - SD^2\Delta,\text{control})}$, with 95% bootstrap CIs (5,000 resamples), expressed relative to the SWC [29,30]; the control group provided the estimate of within-subject and measurement variability.

Supplementary Results

The five-analyte change profile differed between groups (PERMANOVA pseudo-F = 2.35, $R^2 = 0.19$, $P = 0.017$; baseline-adjusted $R^2 = 0.25$, $P = 0.001$; Table S3, Figure S2A). Pairwise, the profile of RBT differed from that of PLY ($P = 0.030$) and, less clearly, from CON ($P = 0.064$), whereas PLY and CON did not differ ($P = 0.10$). The first principal component (34% of variance) loaded negatively on ΔTIMP-1, ΔTIMP-2, ΔCoQ10 and ΔMMP-9 and positively on ΔMMP-2. Given the sample size and the number of analyses, a significant multivariate result does not establish a coordinated biological response.

Inter-individual variability in response, estimated as the SD of individual responses after removing the control-group variability, exceeded the SWC only for MMP-2 (SDIR 12.3 ng/mL in RBT and 10.2 ng/mL in PLY; Table S2, Figure S2B); for TIMP-1, TIMP-2, CoQ10 and MMP-9 the estimates were imprecise and compatible with no heterogeneity beyond measurement and biological noise. Because the pre–post correlation was low for MMP-2 ($r = 0.05$) and several individual MMP-2 concentrations changed by up to 12-fold, these estimates cannot distinguish biological from analytical variability and should not be interpreted as evidence of genuine individual responses.

Table S2. Exploratory bootstrap, equivalence and individual-response analyses for the five analytes (formerly Table 4 of the original submission).

Outcome	Contrast	P(lower) / P(trivial) / P(higher)[a]	Min. equivalence bound (SD units)[b]	TOST P (±0.8 SD)	SDIR (95% CI)[c]	SDIR ÷ SWC
TIMP-1	RBT vs CON	97 / 2 / 1	2.18	0.844	−0.39 (−0.69 to 0.42)	−4.9
	PLY vs CON	99 / 0 / 0	2.21	0.874	−0.37 (−0.68 to 0.48)	−4.7
	RBT vs PLY	32 / 27 / 40	0.93	0.079		
TIMP-2	RBT vs CON	98 / 2 / 0	1.72	0.727	1.65 (−0.91 to 2.22)	6.2
	PLY vs CON	16 / 47 / 37	0.76	0.040	−0.78 (−1.46 to 0.90)	−2.9
	RBT vs PLY	99 / 1 / 0	1.82	0.806		
CoQ10	RBT vs CON	100 / 0 / 0	1.42	0.626	−5.97 (−8.57 to 1.68)	−5.3
	PLY vs CON	33 / 45 / 22	0.62	0.017	−3.59 (−7.82 to 6.17)	−3.2
	RBT vs PLY	95 / 5 / 0	1.41	0.537		
MMP-2	RBT vs CON	34 / 37 / 29	0.69	0.030	12.31 (4.74 to 16.00)	7.2
	PLY vs CON	19 / 41 / 40	0.77	0.043	10.24 (3.09 to 13.64)	6.0
	RBT vs PLY	43 / 39 / 19	0.81	0.053		

Outcome	Contrast	P(lower) / P(trivial) / P(higher)[a]	Min. equivalence bound (SD units)[b]	TOST P (±0.8 SD)	SDIR (95% CI)[c]	SDIR ÷ SWC
MMP-9	RBT vs CON	97 / 3 / 0	1.65	0.597	−0.29 (−0.55 to 0.33)	−3.2
	PLY vs CON	97 / 3 / 0	1.68	0.604	0.23 (−0.49 to 0.57)	2.5
	RBT vs PLY	31 / 39 / 30	0.76	0.041		

[a] Percentage of 4,000 stratified bootstrap resamples of the ANCOVA in which the baseline-adjusted difference (first- minus second-named group) was below −SWC, within ±SWC, or above +SWC (SWC = 0.2 × pooled within-group baseline SD on the analysis scale); descriptive only. [b] Smallest symmetric equivalence bound (pooled within-group baseline SD units) at which TOST would declare equivalence at $P < 0.05$; TOST P is given for bounds of ±0.8 SD, which exclude only very large differences. [c] SD of individual responses in the training group named first, SDIR = $\sqrt{SD^2_{\Delta,training} - SD^2_{\Delta,control}}$; negative values indicate smaller change-score variance than in controls; 95% bootstrap CI (5,000 resamples); expressed in ng/mL for TIMP-2, CoQ10 and MMP-2 and in natural-log units for TIMP-1 and MMP-9; not applicable to RBT vs PLY. CON, control; RBT, elastic-band resistance training; PLY, plyometric training; SWC, smallest worthwhile change; TOST, two one-sided tests.

Table S3. Permutational multivariate analysis of variance (PERMANOVA) of the five-analyte change profile (formerly Table 5).

Comparison	Pseudo-F	R^2	P (permutation)
All groups (raw Δ profile)	2.35	0.19	0.017
All groups (baseline-adjusted Δ profile)	3.33	0.25	0.001
RBT vs CON (raw Δ profile)	2.35	0.15	0.064
PLY vs CON (raw Δ profile)	1.91	0.12	0.105
RBT vs PLY (raw Δ profile)	2.78	0.18	0.030

Change scores (post − pre; log scale for TIMP-1 and MMP-9) of TIMP-1, TIMP-2, CoQ10, MMP-2 and MMP-9 were standardised and Euclidean distances computed between participants; pseudo-F and R^2 follow Anderson (2001) with 9,999 permutations (4,999 for pairwise tests). The baseline-adjusted profile uses change scores residualised on their own baseline value. No correction was applied to the three pairwise tests.

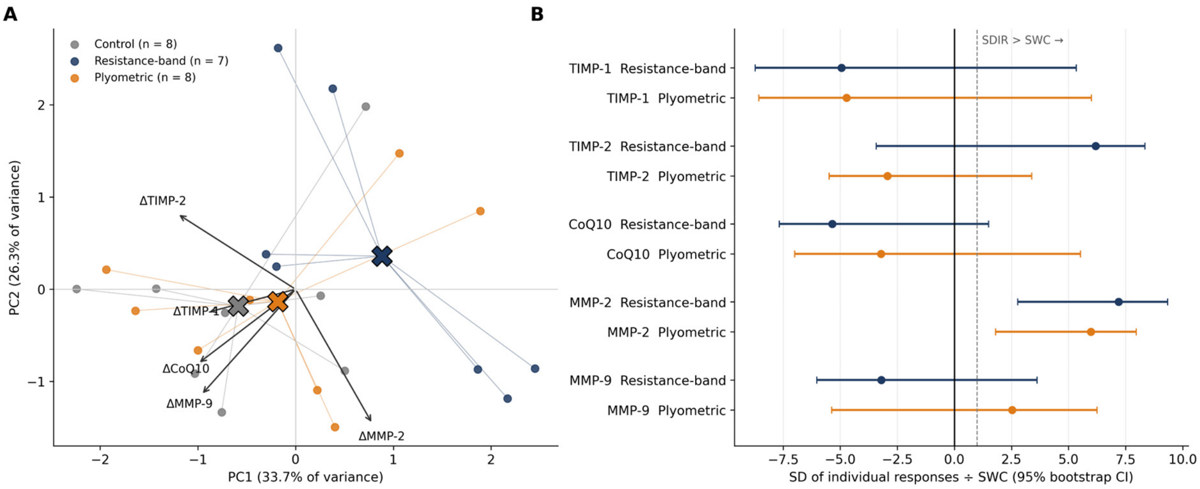

Figure S2. (A) Principal-component analysis of the standardised change scores (Δ = post − pre; log scale for TIMP-1 and MMP-9) of the five analytes in 23 participants. Points are individuals, crosses are group centroids joined to their members by thin lines, and arrows are variable loadings. PERMANOVA on the same Euclidean distances: pseudo-F = 2.35, $R^2 = 0.19$, $P = 0.017$ (baseline-adjusted: $R^2 = 0.25$, $P = 0.001$). (B) Inter-individual variability in the training response, expressed as the SD of individual responses (negative values indicate less variability than in controls) divided by the smallest worthwhile change (0.2 × pooled within-group baseline SD), with 95% bootstrap CIs (5,000 resamples). Dashed line: SDIR = SWC. RBT n = 7, PLY n = 8, CON n = 8 (formerly Figure 4).
